# Tumor in the plantar region: dermatofibrosarcoma protuberans in an infrequent topography^⋆^^⋆⋆^

**DOI:** 10.1016/j.abd.2020.05.002

**Published:** 2020-08-16

**Authors:** Simone Perazzoli, Renan Rangel Bonamigo, Renata Heck, André da Silva Cartell

**Affiliations:** a Sanitary Dermatology Outpatient Clinic, Porto Alegre, RS, Brazil; b Faculty of Medicine, Universidade Federal do Rio Grande do Sul, Porto Alegre, RS, Brazil; c Sanitary Dermatology Service, Secretaria Estadual de Saúde do Estado do Rio Grande do Sul e Santa Casa de Porto Alegre, Porto Alegre, RS, Brazil; d Medical Residence in Pathological Anatomy, Hospital de Clínicas, Porto Alegre, RS, Brazil

**Keywords:** Dermatofibrosarcoma, Immunohistochemistry, Sarcoma

## Abstract

Dermatofibrosarcoma protuberans is a rare mesenchymal tumor; it is locally aggressive and presents high rates of local recurrence. It may present as a nodular or plaque vegetating lesion. It mainly affects the trunk and proximal limbs, being rare in the distal extremities. Biopsy and immunohistochemistry help confirm the diagnosis. The authors report a case of dermatofibrosarcoma protuberans with plantar region involvement, a rare presentation. To the best of the authors’ knowledge, only 11 cases of involvement of the feet were described in the international literature.

## Introduction

Dermatofibrosarcoma protuberans (DFSP) is a mesenchymal neoplasm of slow growth, locally aggressive and with low metastatic potential. It has several presentations and can be characterized as a hardened plaque, nodule, or vegetating lesion.^1^ Local trauma has been described as a potential risk factor for tumor onset.^2^ The most common sites of involvement are the trunk (40%–50%), the proximal limbs (30%–40%), and the head and cervical region (10%–15%). In the literature review, 11 reports of tumors located in the plantar region were retrieved. Due to the rarity of the presentation on the distal limbs, the authors believe that reporting this case is relevant.

## Case report

**Figure 1:**
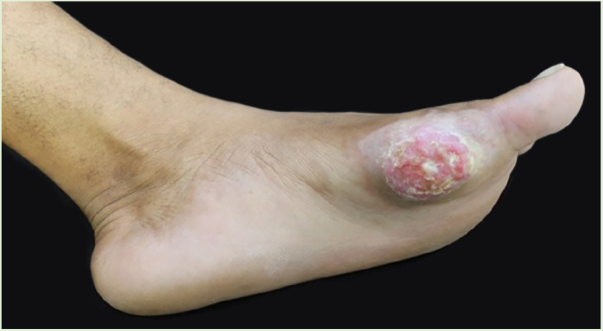
Exophytic and ulcerated tumor lesion.

**Figure 2:**
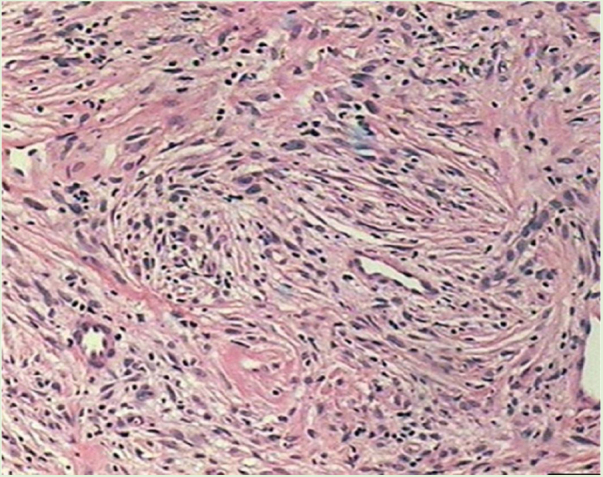
Histopathology: fusocellular proliferation with a focal storiform pattern (Hemathoxylin & eosin ×40).

**Figure 3:**
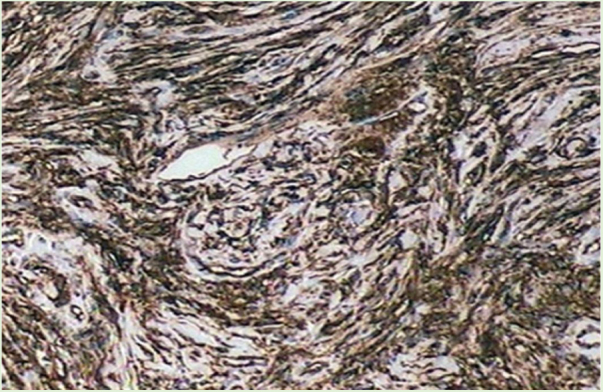
Immunohistochemistry: diffuse positivity for CD34 in spindle cells with storiform pattern.

Male patient, 35 years old, previously healthy, complained of painful lesion on the left plantar region with progressive growth over six months; he denied trauma. On physical examination, he presented an erythematous, exophytic, vegetating, and ulcerated tumor on the first metatarsal joint (Fig. 1). Histopathological examination of the lesion revealed a fusocellular proliferation with a focal storiform pattern (Fig. 2). In the immunohistochemical study, a diffuse positivity for CD34 + cells was observed. The findings were compatible with low-grade fusocellular mesenchymal neoplasia, favoring the diagnosis of DFSP (Fig. 3). Surgical treatment involved amputation of the hallux and part of the left forefoot; no tumor recurrence was observed after four months of post-operative follow-up.

## Discussion

DFSP is a rare mesenchymal tumor with low rates of aggressiveness. It presents slow progression, with high rates of local recurrence and rare cases of distant metastases. An American study conducted between 2000 and 2010 found an incidence of 41 cases in 10 million patients.^3^ Some studies observed a higher incidence in women and black people. It mostly affects the trunk (40%–50%), the proximal limbs (30%–40%), and the head and cervical region (10%–15%).^3^

A literature review published in 2019 described 11 cases of dermatofibrosarcoma that affected the feet. The mean age observed was 41 years; it was more frequent in men than in women (8:3) and the most frequent location of the tumor was the dorsum of the feet. The mean growth period was 3.5 years. The mean DFSP size at diagnosis was approximately 3.5 cm. Clinical diagnosis is difficult given the variety of presentations. Dermoscopy has been described as a useful tool to aid diagnosis.^1^ In a review based on the analysis of 32 dermatofibrosarcomas, the most common features described in dermoscopy were the presence of vessels (81%), followed by a pigment network (78%) and a pinkish background (66%).^3^

Histologically, dermatofibrosarcoma presents monomorphic spindle cells with little atypia and mitotic activity, arranged in irregular and multidirectional (storiform) fascicles. The tumor infiltrates the subcutaneous tissue, creating the characteristic honeycomb pattern. The histological differential diagnoses of other spindle cell tumors are dermatofibroma, malignant fibrous histiocytoma, atypical fibroxanthoma, desmoplastic melanoma, Kaposi's sarcoma, and solitary fibrous tumor.^1^

Immunohistochemistry becomes an important resource for differential diagnosis, as DFSP is positive for CD34 and negative for S 100 protein, factor XIIIA, and desmin.^1^ All the cases described in the literature were tested for CD34 and were positive.^3^

The recommended treatment is lesion excision with 2–3 cm of safety margins.^4^ Involvement of margins is associated with local recurrence.^4^ Another therapeutic option is Mohs micrographic surgery. Management with radiotherapy and imatinib has been described. The use of imatinib is indicated for metastases, local recurrences, as neoadjuvancy, or when the tumor is unresectable.^5^

Attention should be paid to this disease, even in unusual anatomical areas, as the prognosis depends on the early diagnosis and treatment.

## Financial support

None declared.

## Authors’ contributions

Simone Perazzoli: Conception and planning of the study; elaboration and writing of the manuscript; critical review of the literature.

Renan Rangel Bonamigo: Approval of the final version of the manuscript; conception and planning of the study; elaboration and writing of the manuscript; obtaining, analyzing, and interpreting the data; effective participation in research orientation; intellectual participation in propaedeutic and/or therapeutic conduct of studied cases; critical review of the literature; critical review of the manuscript.

Renata Heck: Approval of the final version of the manuscript; conception and planning of the study; elaboration and writing of the manuscript; obtaining, analyzing, and interpreting the data; intellectual participation in propaedeutic and/or therapeutic conduct of studied cases.

André da Silva Cartell: Approval of the final version of the manuscript; conception and planning of the study; intellectual participation in propaedeutic and/or therapeutic conduct of studied cases.

## Conflicts of interest

None declared.
